# Immunoprivileged no more: measuring the immunogenicity of allogeneic adult mesenchymal stem cells

**DOI:** 10.1186/s13287-017-0742-8

**Published:** 2017-12-22

**Authors:** Alix K. Berglund, Lisa A. Fortier, Douglas F. Antczak, Lauren V. Schnabel

**Affiliations:** 1 Department of Clinical Sciences, College of Veterinary Medicine and the Comparative Medicine Institute, North Carolina State University, Raleigh, NC 27607 USA; 2 Department of Clinical Sciences, College of Veterinary Medicine, Cornell University, Ithaca, NY 14853 USA; 3 Baker Institute for Animal Health, Cornell University, Ithaca, NY 14853 USA

**Keywords:** Mesenchymal stem cell, Allogeneic, Immunogenicity, Major histocompatibility complex, Mixed leukocyte reaction, Cytotoxicity, ELISPOT, Microcytotoxicity

## Abstract

**Background:**

Autologous and allogeneic adult mesenchymal stem/stromal cells (MSCs) are increasingly being investigated for treating a wide range of clinical diseases. Allogeneic MSCs are especially attractive due to their potential to provide immediate care at the time of tissue injury or disease diagnosis. The prevailing dogma has been that allogeneic MSCs are immune privileged, but there have been very few studies that control for matched or mismatched major histocompatibility complex (MHC) molecule expression and that examine immunogenicity in vivo. Studies that control for MHC expression have reported both cell-mediated and humoral immune responses to MHC-mismatched MSCs. The clinical implications of immune responses to MHC-mismatched MSCs are still unknown. Pre-clinical and clinical studies that document the MHC haplotype of donors and recipients and measure immune responses following MSC treatment are necessary to answer this critical question.

**Conclusions:**

This review details what is currently known about the immunogenicity of allogeneic MSCs and suggests contemporary assays that could be utilized in future studies to appropriately identify and measure immune responses to MHC-mismatched MSCs.

## Background

Mesenchymal stem cells (MSCs) are currently defined as plastic-adherent cells with a fibroblast-like morphology that are capable of differentiating into bone, cartilage, and fat in vitro and that express a defined set of surface markers, which vary slightly by species [1, 2]. The origin of MSCs in vivo is controversial, but there is evidence to support that MSCs are a type of pericyte or adventitial cell [3, 4]. The multipotent properties of MSCs led to initial conclusions that these cells could be used clinically to repair or regenerate injured tissues [5], and animal studies supported that MSCs provided a therapeutic benefit [6]. However, MSCs have poor engraftment rates [7, 8] and there is little evidence to suggest that the primary function of MSCs is to differentiate into new tissue in vivo [9], questioning the relevance of differentiation to the therapeutic properties of MSCs when injected in a naive state. Tri-lineage differentiation assays may still be important in some cases for confirming that the cells used in studies are MSCs, since MSCs and fibroblasts have similar morphology and phenotype [10].

Secretion of paracrine factors is now recognized as the primary mechanism by which MSCs promote a regenerative environment conducive to healing with healthy tissue [11], although cell-to-cell contact has also been shown to be important under some conditions [12, 13]. MSCs home to sites of inflammation where they secrete a variety of soluble factors including growth factors, cytokines, and chemokines [14]. In-vivo studies have demonstrated that MSC therapy promotes angiogenesis and growth and differentiation of local progenitor cells, prevents fibrosis and apoptosis, attracts immune cells to the site of injury, and modulates immune responses [14–17]. As engraftment appears to be unnecessary for the therapeutic effect, exogenous MSCs likely need to persist through the initial inflammatory phase and into the repair and remodeling phase of tissue healing to have a full therapeutic effect. Adult MSCs, which are obtained from the bone marrow, peripheral blood, or adipose tissue of patients, are currently being investigated in over 450 clinical trials to treat numerous diseases including musculoskeletal diseases, degenerative and traumatic neurological diseases, and immune-mediated diseases [18]. MSC therapy has been effective at treating several animal models of disease [19, 20] and shown success in human clinical trials [18]. The therapeutic benefits of MSC therapy demonstrated in preclinical trials has not translated to success in every human clinical trial, however, and the use of allogeneic versus autologous MSC therapy is one factor that may contribute to the differences in efficacy seen in some clinical trials [21, 22].

In-vitro expansion of MSCs prior to clinical use can take several weeks to obtain enough cells for administration, resulting in loss of stemness; the age and disease state of the patient can also negatively affect the quality of the cells [23, 24]. Adult allogeneic MSC therapy is particularly attractive as it allows for immediate treatment with quality cells at the time of injury or diagnosis. In early studies, researchers discovered that allogeneic MSCs were capable of inhibiting the proliferation of major histocompatibility complex (MHC)-mismatched lymphocytes in mixed leukocyte reactions (MLR) in vitro [25]. MSCs produce a variety of immunomodulatory cytokines including transforming growth factor-β1, indoleamine 2,3-dioxygenase, inducible nitric oxide synthase, and prostaglandin E_2_, which contribute to the ability of MSCs to modulate immune responses [14]. This discovery initially indicated that MSCs were “immunoprivileged” and were subsequently promoted as safe to use in allogeneic settings without concern for immune rejection [25].

Although allogeneic MSC therapy is generally regarded as safe [26], there have been several reports of adverse clinical events including increased synovial cellularity and total nucleated cell counts following intra-articular injection of allogeneic MSCs in equine models [27, 28]. Most studies do not characterize if allogeneic donor MSCs and recipients are MHC-matched or MHC-mismatched, nor do they investigate if the MSCs induce immune responses and are rejected. Furthermore, few studies have compared allogeneic versus autologous MSC therapy using cells of comparable quality to determine if there is a difference in efficacy for tissue healing or disease outcome and if those differences correlate or not with immune rejection of allogeneic MSCs. In order to fully understand the potential of allogeneic MSC therapy, further investigation into the immune responses towards allogeneic cells and if immune responses affect the therapeutic outcome of MSC therapy are warranted.

The purposes of this review are to outline what is currently understood about immune responses to adult allogeneic MSCs and to describe contemporary assays that could be utilized in future preclinical studies and clinical trials to appropriately identify and measure immune responses to allogeneic MSCs. By gaining a better understanding of how and under what circumstances a recipient immune system responds to allogeneic MSCs, researchers can develop strategies to improve allogeneic MSC efficacy and ensure safety.

## In vivo immunogenicity of allogeneic MSCs

The few published studies that controlled for MHC haplotype of donors and recipients and assessed immune responses following injection of MSCs support that adult MHC-mismatched MSCs are not immune privileged. In multiple animal models, bone marrow-derived MHC-mismatched MSCs induced both cell-mediated and humoral immune responses in vivo and were subsequently rejected (Table 1) [29–35]. These studies provided valuable information about how the immune system responds to MHC-mismatched MSCs, although the clinical implications of MSC rejection by the recipient immune system are still not entirely clear.

**Table 1:** In-vivo studies with MHC controls and immune response analysis

Author	Species	Cell-mediated	Humoral	In vivo rejection	Methods used
Eliopoulos and Stagg, 2005 [29]	Mouse	+		+	In-vivo cytotoxicity
Nauta et al., 2006 [30]	Mouse	+		+	In-vivo cytotoxicity
Badillo et al., 2007 [31]	Mouse	+	+		Ex-vivo MLR, allograft rejection
Poncelet et al., 2007 [32]	Pig	+	+		Ex-vivo MLR, CDC
Zangi et al., 2009 [33]	Mouse	+	+	+	Allograft rejection, in-vivo imaging
Isakova et al., 2014 [34]	Rhesus Macaques	+			In-vitro cytotoxicity
Pezzanite et al., 2015 [35]	Horse		+		CDC

*CDC* complement-dependent cytotoxicity, *MHC* major histocompatibility complex, *MLR* mixed leukocyte reaction

Cell-mediated responses to MSCs are induced when T cells become activated following recognition of foreign donor MHC molecules expressed on the surface of the MSCs. Significant increases in circulating T cells and natural killer cells were detected in rhesus macaques as early as 10 days after intracranial injection with MHC-mismatched MSCs, but not those injected with autologous MSCs [34]. Cytotoxic peripheral blood leukocytes (PBLs) capable of lysing donor MSCs were also found in macaques that received MHC-mismatched MSCs but not macaques that received autologous MSCs. In this study, the degree of MHC I and MHC II mismatch between donor and recipient correlated with the magnitude of the immune response, supporting that the immune response against donor MSCs was MHC-specific. This study also demonstrated that injection of MHC-mismatched MSCs into a relatively immune privileged area like the central nervous system (CNS) did not prevent immune responses against the cells. Studies in mice and pigs have established that MHC-specific memory lymphocytes are generated in response to MHC-mismatched MSCs [29, 31–33]. Mice injected intravascularly with MHC-mismatched MSCs had significant increases in CD4^+^ and CD8^+^ splenocytes with a memory phenotype (CD122^+^CD44^+^CD62L^low^), but not mice injected with MHC-matched MSCs [33]. In separate studies where mice were injected intraperitoneally and a pig injected intracardiacally with MHC-mismatched MSCs, responder lymphocytes showed accelerated proliferation in an ex-vivo MLR when exposed to stimulator cells of the same MHC haplotype as donors, demonstrating the presence of MHC-specific memory lymphocytes [31, 32]. The formation of memory immune cells in recipients of MHC-mismatched MSCs is important since immunologic memory can lead to accelerated rejection of allogeneic cells upon reinjection. Collectively, these studies indicate that, regardless of the species or route of administration, recipient lymphocytes are sensitized to mismatched MHC molecules expressed by donor MSCs and differentiate into MHC-specific effector and memory cells.

Pre-existing antibodies crossreactive for donor MHC molecules or alloantibodies produced following activation of B cells by cognate alloantigens can also contribute to rejection of allogeneic cells. A significant increase in total serum immunoglobulin (Ig)G was reported in rhesus macaques injected with MHC-mismatched MSCs, but not in macaques injected with autologous MSCs [34]. Alloantibodies have also been detected in mice, pigs, and horses injected with MHC-mismatched MSCs [31, 32, 35]. Horses injected intradermally with MHC-mismatched MSCs generated cytotoxic anti-MHC I alloantibodies as early as 7 days postinjection, while a control horse injected with MHC-matched MSCs did not [35, 36]. Anti-MHC antibodies and alloreactive T cells have been detected following exposure to unrelated proteins [37–39] so it is possible for recipients to be primed against allo-MHC molecules and mount antibody responses quickly against allogeneic MSCs after a single injection. Two recent human MSC clinical trials monitored patients for alloantibody production and found that while the majority of patients do not develop significant alloantibody after injection with allogeneic MSCs, a minority of patients do develop alloantibodies [40, 41]. It is possible that induction of alloantibodies by allogeneic MSC therapy is correlated to the degree of MHC-mismatch between donor and recipient and further supports that MHC haplotyping of donors and recipients be performed. The health and immune status of recipients may also be important and should be fully disclosed in future clinical trials. Hyperacute rejection-like symptoms have not been reported in human patients who receive allogeneic MSC therapy, but further investigation into alloantibodies induced by MSCs is warranted to protect patients who may receive multiple injections of allogeneic MSCs [42] or patients who may have been previously sensitized to human leukocyte antigens (HLAs) from a pregnancy, blood transfusion, or an organ transplantation.

In-vivo rejection of MSCs has been measured both directly using bioluminescent imaging and in-vivo cytotoxicity assays and indirectly by measuring hematocrit following injection with erythropoietin-expressing MSCs. In each of these studies, MHC-mismatched MSCs survived for a significantly shorter period of time than MHC-matched MSCs in immunocompetent mice and were rejected more quickly in previously sensitized animals [29, 30, 33]. MHC-mismatched MSCs did persist longer than MHC-mismatched fibroblasts, however, supporting that they are still immunomodulatory in vivo [33]. While the immunomodulatory properties of MSCs may improve survival compared to non-immunomodulatory cells such as fibroblasts, recipient immune responses appear to limit survival of MHC-mismatched MSCs compared to MHC-matched MSCs. Although large animal studies in macaques, horses, and pigs have measured immune responses that could potentially lead to the rejection of MSCs, in-vivo rejection has currently only been measured using mouse models. While it is likely that a similar phenomenon may occur in large animals and humans, it is currently unknown in these species how long MHC-mismatched MSCs survive following injection, how quickly they are rejected, or if rejection is primarily due to cell-mediated or humoral immune responses. The answers to these questions may help with the development of targeted strategies to limit the rejection and retain the therapeutic window of efficacy for MHC-mismatched MSCs in vivo.

## Methods to measure immune responses

A number of standard immunological assays and techniques are available to measure the immunogenicity of MSCs. For these assays, the MHC haplotype of donors, recipients, stimulators, and responders should be determined to understand if donor or stimulator MSCs are full or partial mismatches to recipients or responder cells. Control cells should include donor or target cells of the same MHC haplotype as recipients or responders to control for MHC-specific immune responses. Modified one-way in-vitro MLRs, where responder splenocytes or PBLs are cocultured with stimulator allogeneic MSCs, have traditionally been used to measure the immunogenicity of MSCs, but several studies have demonstrated that in-vitro MLR assays are poor predictors of in-vivo immunogenicity [30, 32, 35, 43]. It is likely that the high cell concentrations and cytokine levels relative to physiologic levels in an MLR account for discrepancies in MSC in-vitro and in-vivo immunogenicity. The immunomodulatory functions of MSCs can be measured using traditional MLRs, where responder and stimulator splenocytes or PBLs are cocultured with MSCs, but the ability of allogeneic MSCs to suppress T-cell proliferation does not correlate with the in-vivo immunogenicity of allogeneic MSCs either [29, 31, 33, 34].

**Fig. 1:**
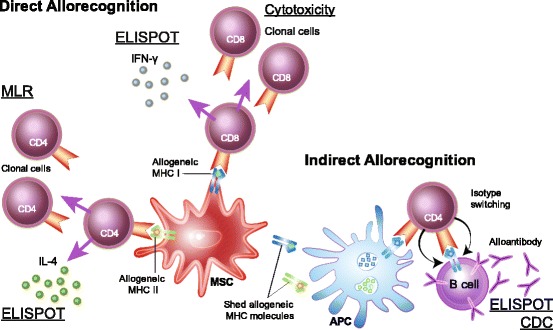
In-vivo immune responses to MHC-mismatched MSCs and corresponding assays. Following injection of MHC-mismatched MSCs in vivo, allogeneic MHC I molecules are directly recognized by alloreactive CD8^+^ T cells, which induces secretion of interferon (IFN)-γ and clonal expansion of cytotoxic T cells. IFN-γ secretion by T cells restimulated with donor allogeneic MHC I molecules can be measured using an ELISPOT. Effector function of cytotoxic T cells specific for donor allogeneic MHC I molecules can be measured using cytotoxicity assays. Allogeneic MHC II molecules are directly recognized by alloreactive CD4^+^ T cells, which induces secretion of IL-4 or IFN-γ and clonal expansion of helper T cells. IL-4 secretion by T cells restimulated with donor allogeneic MHC II molecules can be measured by ELISPOT. Expansion of MHC-specific CD4^+^ T cells can be detected using an ex-vivo MLR. Allogeneic MHC molecules can be shed into the environment where they are processed and presented to lymphocytes by APCs. Following activation by allogeneic MHC peptides, B cells can produce alloantibodies with the support of CD4^+^ T cells activated by indirect allorecognition. Alloantibodies can be detected by ELISPOT or complement-dependent cytotoxicity assays. *APC* antigen-presenting cell, *CDC* complement-dependent cytotoxicity, *ELISPOT* enzyme-linked immunospot, *IL-4* interleukin-4, *MHC* major histocompatibility complex, *MLR* mixed leukocyte reaction, *MSC* mesenchymal stem cell

Measuring heat, swelling, or the infiltration of immune cells or lack thereof into the site where MSCs were injected or the tissue of interest is also not sufficient to determine if donor MSCs have induced an immune response. The absence of a local immune response does not rule out a systemic response (for example, in the spleen where MSCs may home following injection [44]) and does not measure if there is an MHC-specific response. Overall changes in peripheral blood lymphocyte counts also do not indicate if there is a targeted immune response to MHC-mismatched MSCs. Similarly, overt clinical signs such as fever or anaphylaxis have not been found to correlate with immune responses or rejection of allogeneic MSCs [35, 45]. When possible, functional assays should be performed to determine the type of immune responses and evaluate the potential implications for clinical therapy. The in-vivo immune response to MHC-mismatched MSCs and appropriate assays for detecting each response are depicted in Fig. 1. When testing in-vivo immunogenicity, assays should be performed prior to injection of donor MSCs in humans and large animals or in untreated control animals to measure baseline immune responses. Testing at multiple time points after administration is also preferable for measuring the kinetics of the immune response. Appropriate assays for detecting immune responses against MSCs are summarized in Table 2.

**Table 2:** Assays for measuring cell-mediated and humoral immune responses against allogeneic MSCs

Assay	Effector cell	Target cell	Immune function measured	Outcome measurement
Ex-vivo MLR	Lymphocytes (primarily CD4^+^)	Splenocytes/lymphocytes	Cell-mediated	T-cell proliferation
Cytotoxicity	Cytotoxic T lymphocytes	MSCs	Cell-mediated	Target cell death
ELISPOT	T or B cell	Splenocytes/lymphocytes	Cell-mediated or humoral	Cytokine secretion
Antibody-dependent CDC	B cell	Lymphocytes or MSCs	Humoral	Target cell death
In vivo imaging		MSCs		MSC survival

*CDC* complement-dependent cytotoxicity, *ELISPOT* enzyme-linked immunospot, *MLR* mixed leukocyte reaction, *MSC* mesenchymal stem cell

### Cell-mediated functional assays

Ex-vivo MLRs can be useful for estimating sensitization of recipient T cells to donor MHC molecules postadministration with allogeneic MSCs. Splenocytes or PBLs from recipients are collected and used as responders in a standard one-way MLR using stimulator splenocytes or PBLs of the same MHC haplotype as the MSC donor [31]. Proliferation of responder PBLs indicates immune cell recognition of allo-MHC molecules and subsequent activation. Accelerated proliferative responses in an ex-vivo MLR following administration of MSCs demonstrate differentiation and activation of donor MHC-specific memory lymphocytes.

Interferon (IFN)-γ and interleukin (IL)-4 enzyme-linked immunospots (ELISPOTs) can measure functional responses of T cells upon restimulation with donor MHC molecules as well as predict the in-vivo immunogenicity. Similar to the ex-vivo MLR, following in-vivo administration of MSCs, recipient splenocytes or PBLs can be isolated and restimulated using splenocytes or PBLs of the same MHC haplotype as donor MSCs. Secretion of IFN-γ or IL-4 above baseline indicates expansion of CD8^+^ or CD4^+^ effector and memory cells against donor MSCs [46]. Preformed T-cell responses measured using ELISPOTs accurately predict graft rejection in organ transplantation cases [47] and may be able to predict the in-vivo cell-mediated immunogenicity of donor MSCs. Enzyme-linked immunosorbent assays (ELISAs) can also measure changes in cytokine secretion from restimulated recipient splenocytes or PBLs, but cannot measure the frequency of MHC-specific immune cells.

Cytotoxicity assays can be used to measure direct lysis of MSCs by MHC-specific cytotoxic T lymphocytes (CTLs). As MSCs inhibit formation of CTLs in MLRs [48], effector cells should be induced in a standard MLR with splenocyte or PBL stimulator cells or in vivo [49]. If MSCs are administered in vivo the cytotoxicity of MSCs of the same MHC haplotype as the donor by splenocytes or PBLs can be compared against baseline cytotoxicity to measure expansion of CTLs. Increases in cytotoxicity above baseline indicates that differentiation of T cells into MHC-specific effector and memory CTLs has occurred and that the immune system is capable of rejecting the donor MSCs. In-vitro cytotoxicity assays can be performed using a standard chromium 51 assay [49] or newer flow cytometry-based assays [50]. In-vivo cytotoxicity assays have also been described and can be utilized in small animal models [51].

### Humoral assays

Several assays are available for detecting alloantibodies and identifying the specificity and function of alloantibodies. Microcytotoxicity assays, also called lymphocytotoxicity assays, were originally developed for tissue typing, but can also be used to detect cytotoxic anti-MHC alloantibodies in serum. Standard one- or two-stage microcytotoxicity assays use eosin or fluorescent dye to detect antibody-mediated complement-dependent cytotoxicity (CDC) following incubation of sera from animals injected with MHC-matched or MHC-mismatched MSCs with donor PBLs or MSCs and rabbit complement [35, 52]. Flow cytometry-based CDC assays have also been utilized with MSCs [32]. Due to the simplicity of these assays, CDC assays can be performed using serum and target cells from nontraditional model organisms that lack the commercial reagents available for humans.

Donor MSC-specific antibodies can also be detected by incubating donor MSCs or PBLs of the same MHC haplotype with sera from recipients and staining with anti-IgG or anti-IgM secondary antibodies [31]. Single antigen bead (SAB) assays, ELISPOTs, and HLA-tetramers have also been used to screen human sera for MHC-specific alloantibodies [53], and commercial kits are readily available. However, these assays do not determine the functionality of the alloantibodies.

### Imaging

In-vivo imaging can also be used to track the survival of injected MSCs. MSCs from transgenic mice that constitutively express luciferase or fluorescent proteins allow for long-term tracking of cells and estimations of survival in vivo [33]. For larger animal models, where MSCs cannot be imaged in vivo by bioluminescence, iron oxide-labeled MSCs have been tracked via magnetic resonance imaging (MRI) [54]. The disadvantage to labeling cells with iron oxide is that the signal will persist even after the MSCs have died or have been phagocytosed [55]. Labeling of cells with membrane dyes also allows for identifying remaining transplanted MSCs on histology, but the cells cannot be tracked over time [54]. Imaging alone cannot detect or assess immune responses, but when used with the other functional assays described above it can help determine the effects and kinetics of an immune response on MSC survival.

## Conclusions

Allogeneic MSC therapy holds significant promise for treating numerous diseases, but further studies are needed to assess the potential of allogeneic MSCs for widespread clinical use. In-vitro and in-vivo studies designed with appropriate MHC controls and thorough immune response analyses will help answer under what conditions immune responses to allogeneic MSCs occur and if these immune responses affect the safety and efficacy of MSC therapy. Additionally, as animal studies support that allogeneic MSCs are rejected in vivo, strategies to reduce the immunogenicity and increase the ability of MSCs to avoid immune responses should be investigated to enhance the survival of allogeneic MSCs.

## Abbreviations

CDC: Complement-dependent cytotoxicity
CTL: Cytotoxic T lymphocyte
ELISPOT: Enzyme-linked immunospot
HLA: Human leukocyte antigen
IFN: Interferon
Ig: Immunoglobulin
IL: Interleukin
MHC: Major histocompatibility complex
MLR: Mixed leukocyte reaction
MRI: Magnetic resonance imaging
MSC: Mesenchymal stem cell
PBL: Peripheral blood leukocyte
